# Inexhaustible hair-cell regeneration in young and aged zebrafish

**DOI:** 10.1242/bio.012112

**Published:** 2015-05-22

**Authors:** Filipe Pinto-Teixeira, Oriol Viader-Llargués, Elen Torres-Mejía, Melissa Turan, Estela González-Gualda, Laura Pola-Morell, Hernán López-Schier

**Affiliations:** Laboratory of Sensory Cell Biology & Organogenesis, Centre for Genomic Regulation, Dr. Aiguader 88, 08003 Barcelona, Spain

**Keywords:** Adult, Hair cells, Lateral line, Notch, Regeneration, Self organization

## Abstract

Animals have evolved two general strategies to counter injury and maintain physiological function. The most prevalent is protection by isolating vital organs into body cavities. However, protection is not optimal for sensory systems because their external components need to be exposed to the environment to fulfill their receptive function. Thus, a common strategy to maintain sensory abilities against persistent environmental insult involves repair and regeneration. However, whether age or frequent injuries affect the regenerative capacity of sensory organs remains unknown. We have found that neuromasts of the zebrafish lateral line regenerate mechanosensory hair cells after recurrent severe injuries and in adulthood. Moreover, neuromasts can reverse transient imbalances of Notch signaling that result in defective organ proportions during repair. Our results reveal inextinguishable hair-cell regeneration in the lateral line, and suggest that the neuromast epithelium is formed by plastic territories that are maintained by continuous intercellular communication.

## INTRODUCTION

**Fig. 1.**
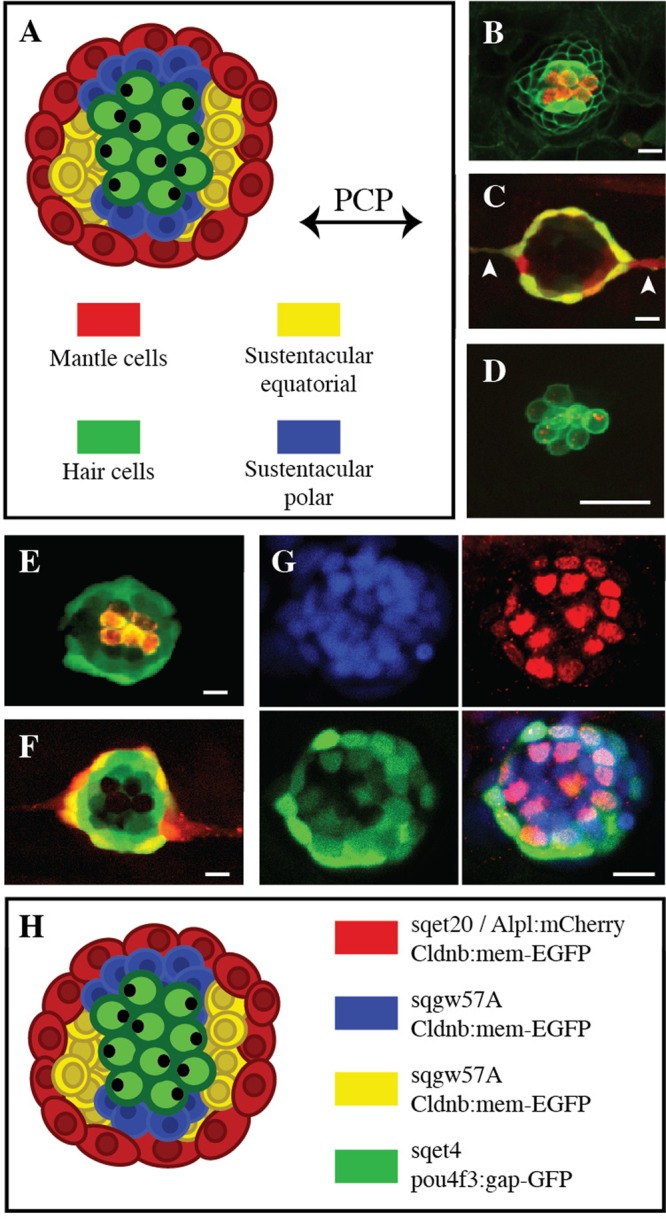
**Neuromast organization and transgenic zebrafish lines.** (A) Scheme of a neuromast depicting different cell types and axes of symmetry. (B-F) Confocal image of a larval neuromast of (B) *Tg[Cldnb:mem-EGFP;ET(krt4:EGFP)sqet4] (green)*, incubated in the vital dye DiAsp to reveal functional hair cells (red), (C) *Tg[Alpl:mCherry (red) ; ET(krt4:EGFP)sqet20 (green)]* in which white arrowhead point to interneuromast cells, (D) *Tg[pou4f3:gap43-GFP (green) ; pou4f3:Ribeye-Kusabira (red)],* (E) *Tg[ET(krt4:EGFP)sqgw57A] (green)* labeled with DiAsp (red), (F) *Tg[ET(krt4:EGFP)sqgw57A (green) ; Alpl:mCherry (red)],* (G) *Tg[ET(krt4:EGFP)sqgw57A]* (green) immunostained for Sox-2 (red) and incubated with the nuclear dye DAPI (blue). (H) Overview of a neuromast with the different cell types and transgenic markers. Scale bars=10 µm.

Sensory receptors are the interphase between the environment and the nervous system. Vertebrates are generally able to repair sensory organs, but mammals cannot regenerate the hair cells of their inner ear (Burns and Corwin, 2013; Groves et al., 2013; Rubel et al., 2013). Consequently, ototoxic antibiotics, anti-neoplastic therapies or high levels of sound cause irreversible hearing loss and balance disorders (Chen and Fechter, 2003; Huth et al., 2011; Langer et al., 2013). Hair cells in fishes and amphibians occurring in the ear and the lateral line are also susceptible to damage by overstimulation or by drug-induced ototoxicity (Harris et al., 2003; Jiang et al., 2014; López-Schier and Hudspeth, 2006; Ma et al., 2008; Millimaki et al., 2010; Schuck and Smith, 2009; Steiner et al., 2014). However, these aquatic vertebrates can rapidly regenerate hair cells (Behra et al., 2009; Hernández et al., 2007; Williams and Holder, 2000). The superficial lateral line of zebrafish is composed by a collection of neuromasts that are formed by a simple circular epithelium of approximately 60 cells. Some 16–20 mechanosensory hair cells occupy the center of the organ, whereas the remainder are two types of non-sensory supporting cells: around 30 sustentacular cells intermingle with hair cell, and fewer than 10 mantle cells outline the organ (Fig. 1A). Interneuromast cells connect each organ. Lateralis hair cells are born in pairs from the terminal mitotic division of unipotent hair-cell progenitors (UHCPs). The neuromast has a stereotypical symmetry formed by one axis of planar cell polarity and by the position of functionally distinct non-sensory equatorial and polar areas (Fig. 1A). Equatorial cells are under sustained Notch signaling, which restricts the development of UHCPs to permissive polar areas with low Notch (Wibowo et al., 2011). Stem cells have not been described in neuromasts, and whether the regenerative capacity of neuromasts diminishes with age or after recurrent damage remains unknown.

## RESULTS AND DISCUSSION

### The *ET(krt4:EGFP)sqgw57A* transgenic line highlights Sox-2^+^ cells in neuromasts

To assay neuromast architecture we acquired a collection of fluorescent transgenic lines with complementary expression patterns. As shown previously, the green-fluorescent line *Tg[Cldnb:mem-EGFP]* highlights the whole neuromast and the interneuromast cells, and weakly the peridermal cells (Fig. 1B) (Haas and Gilmour, 2006; López-Schier and Hudspeth, 2006). The *Tg[ET(krt4:EGFP)sqet20]* line marks interneuromast cells and highlights the equatorial areas (Fig. 1C, supplementary material Fig. S1) (López-Schier and Hudspeth, 2006; Parinov et al., 2004), whereas the red-fluorescent *Tg[Alpl:mCherry]* is expressed homogeneously in the peripheral cells of the neuromast and in interneuromast cells (Fig. 1C, supplementary material Fig. S1) (Steiner et al., 2014). *Tg[ET(krt4:EGFP)sqet4]* expresses EGFP in the UHCPs and hair cells (Fig. 1B) (López-Schier and Hudspeth, 2006; Parinov et al., 2004; Wibowo et al., 2011), and the *Tg[pou4f3:gap43-GFP]* only marks the hair cells (Fig. 1D) (Xiao et al., 2005). Next, we established a new transgenic line called *Tg[ET(krt4:EGFP)sqgw57A]* to better characterize hair-cell regeneration *in vivo*. It was generated by the genomic insertion of a gene-trapping vector carrying a green-fluorescent protein (Kondrychyn et al., 2011). We found that *Tg[ET(krt4:EGFP)sqgw57A]* expresses EGFP in Sox-2^+^ cells, but not in interneuromast cells or hair cells (Fig. 1E-G). Sox-2 is a transcription factor at the apex of the gene-expression cascade that establishes sensory competence in the neuroepithelium at the earliest stages of hair-cell development (Kiernan et al., 2005; Millimaki et al., 2010; Neves et al., 2013). In the zebrafish lateral line and inner ear, cells expressing Sox-2 are the source of hair-cell progenitors (Hernández et al., 2007; Millimaki et al., 2010). Therefore, *Tg[ET(krt4:EGFP)sqgw57A]* is likely to highlight the cells that will be canalized to a UHCPs fate in permissive polar areas. This comprehensive collection of transgenic lines allows the unambiguous visualization of cell identity, distribution, and number in neuromasts (Fig. 1H).

### Hair cells regenerate efficiently in larval, juvenile and adult zebrafish

**Fig. 2.**
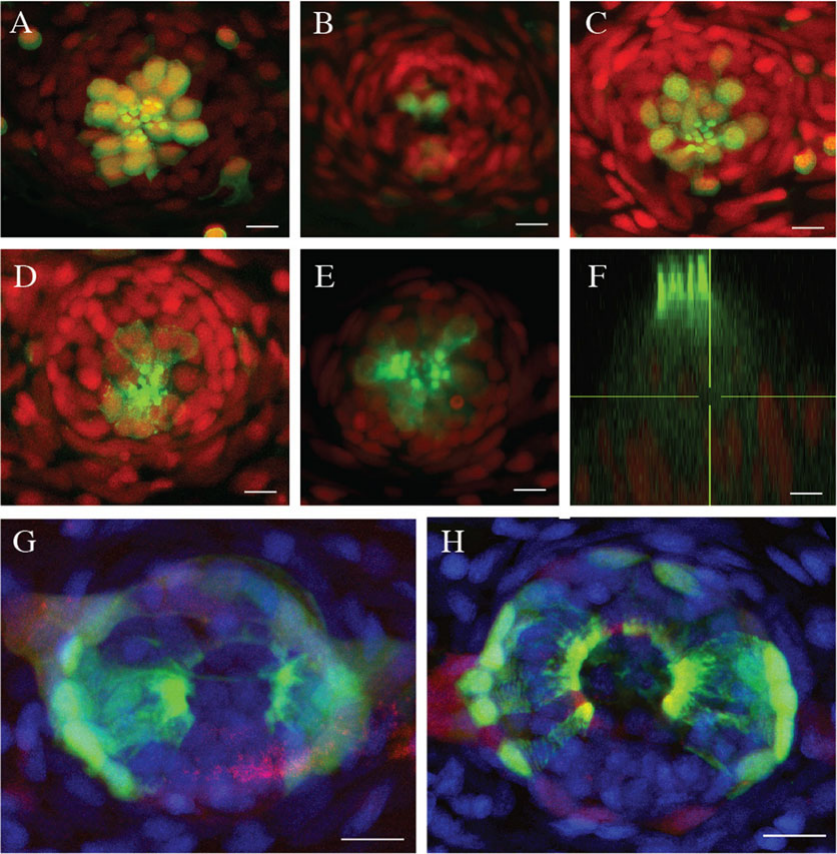
**Efficient hair-cell regeneration in adult zebrafish.** (A-C) Maximal projection of confocal images from *Tg[ET(krt4:EGFP)sqet4]* transgenics (green) showing neuromasts of the caudal fin of a 2-year old fish stained with DAPI (red) (A) before neomycin-treatment, (B) 2 h after treatment, and (C) 72 h after treatment. (D-E) Neuromast of an adult fish from *Tg[myo6b:actb1-EGFP]* transgenics (green) stained with DAPI (red) showing (D) hair cells controls, and (E) in neomycin-treated fish after hair-cell regeneration. (F) XZ profile of the same neuromast in E showing the apicobasal polarization of the regenerated hair cells (green). (G-H) Neuromast of an adult fish from *Tg[Alpl:mCherry (red) ; ET(krt4:EGFP)sqet20 (green)]* transgenics stained with DAPI (blue), showing (G) epithelial geometry in control fish and (H) in neomycin-treated fish at 72 hpt. In all cases, *N*=8 neuromasts from 2 animals. Scale bars=10 µm.

A single treatment with the ototoxic aminoglycoside antibiotic neomycin readily ablates every functional hair cell in the superficial lateral line of the zebrafish larva (Harris et al., 2003; López-Schier and Hudspeth, 2006; Pinto-Teixeira et al., 2013). Subsequently, neuromasts enter a regenerative process that is largely complete 72 hours post (neomycin) treatment (hpt) (Ma et al., 2008; Wibowo et al., 2011). To assess hair-cell regeneration in older animals, we treated three different transgenic lines at juvenile (3-month old) and adult (1- and 2-year old) stages with neomycin. In all cases, hair-cell regeneration occurred within 72 hpt (Fig. 2A-C, and data not shown). Using 1-year old adult fish in which the *Tg[myo6b:actb1-EGFP]* transgene reveals the apical hair bundle of the hair cells (Fig. 2D-F), and 6-month old *Tg[Alpl:mCherry ; ET(krt4:EGFP)sqet20]* that shows neuromast geometry (Fig. 2G-H), we found that cell polarity and epithelial architecture were comparable between controls and neomycin-treated samples 72 hpt. Thus, neuromasts are endowed with invariant and enduring regenerative capacity, which may have evolved for fish to maintain life-long sensory ability despite persistent environmental insult (Ciba-Foundation, 1991).

### Hair-cell regeneration is unaffected by the recurrent and frequent loss of hair cells

**Fig. 3.**
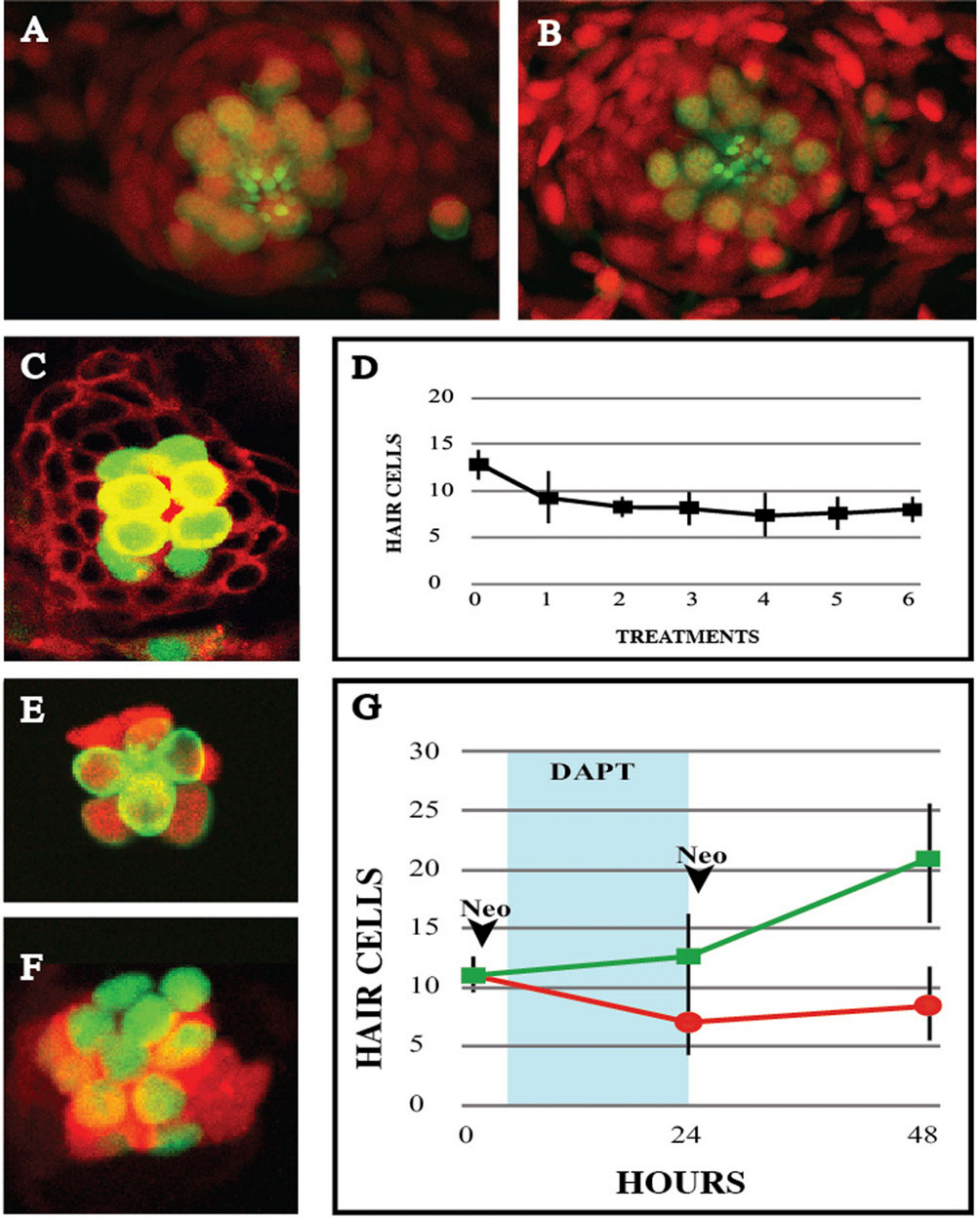
**Hair-cell regeneration after recurrent damage.** (A-B) Confocal images of a *Tg[ET(krt4:EGFP)sqet4] (green)* larval neuromast labeled with DAPI (red) showing hair cells (A) before neomycin treatment and (B) 72 h after the 6th treatment. (C) Image of a *Tg[ET(krt4:EGFP)sqet4] (green)* larval neuromast counterstained for cellular membranes (red) 24 hpt, with 8 hair cells. (D) Graph depicting the number of hair cells per neuromast 24 h after each neomycin treatment, over the course of 6 consecutive treatments. (E-F) A neuromast from a neomycin-treated larva *Tg[Atoh1a:dTomato (red) ; ET(krt4:EGFP)sqet4 (green)]* without (E), and with Notch inhibition with DAPT (F), showing more numerous hair cells and stronger and broader Atho1a expression. (G) Graph showing number of hair cells per neuromast 24 h after two neomycin treatments with (green) and without (red) inhibition of Notch. DAPT incubation period is shadowed in blue. Results are mean±s.d. Time points: 0 h *N*=5 neuromasts (5 animals), 24 h *N*=8 neuromasts (8 animals), 48 h *N*=4 neuromasts (4 animals).

After treatment with neomycin, the first hair cells appear around 8 hpt, with a sequential production of pairs of hair cells up to 20 by 72 hpt (Ma et al., 2008; Wibowo et al., 2011; Lush and Piotrowski, 2014). We hypothesized that the fast onset of regeneration can be explained by the presence of a subpopulation of “primed” cells that are quickly routed towards a UHCP fate. Therefore, a frequent and long sequence of hair-cell ablations should deplete the epithelium from primed cells, leading to regenerative decline. To test this idea we subjected 2-year old transgenics *Tg[ET(krt4:EGFP)sqet4]* to six consecutive neomycin treatments with intervening 24-h periods of rest between treatments to allow partial regeneration. Hair cells regenerated efficiently after the sixth injury cycle (Fig. 3A-B). To assess the temporal profile of regeneration, we subjected *Tg[ET(krt4:EGFP)sqet4]* larvae to six consecutive hair-cell ablations with neomycin and counted hair cells 24 h after each treatment. Neuromasts showed invariable hair-cell regeneration after each ablation (Fig. 3C-D). Thus, to maintain this inexhaustible regenerative capacity at constant kinetics, each organ must persistently produce at least 4 UHCPs per day, representing over 10% of the originating Sox-2^+^ cells.

### Atoh1a is transiently and broadly expressed in the epithelium

**Fig. 4.**
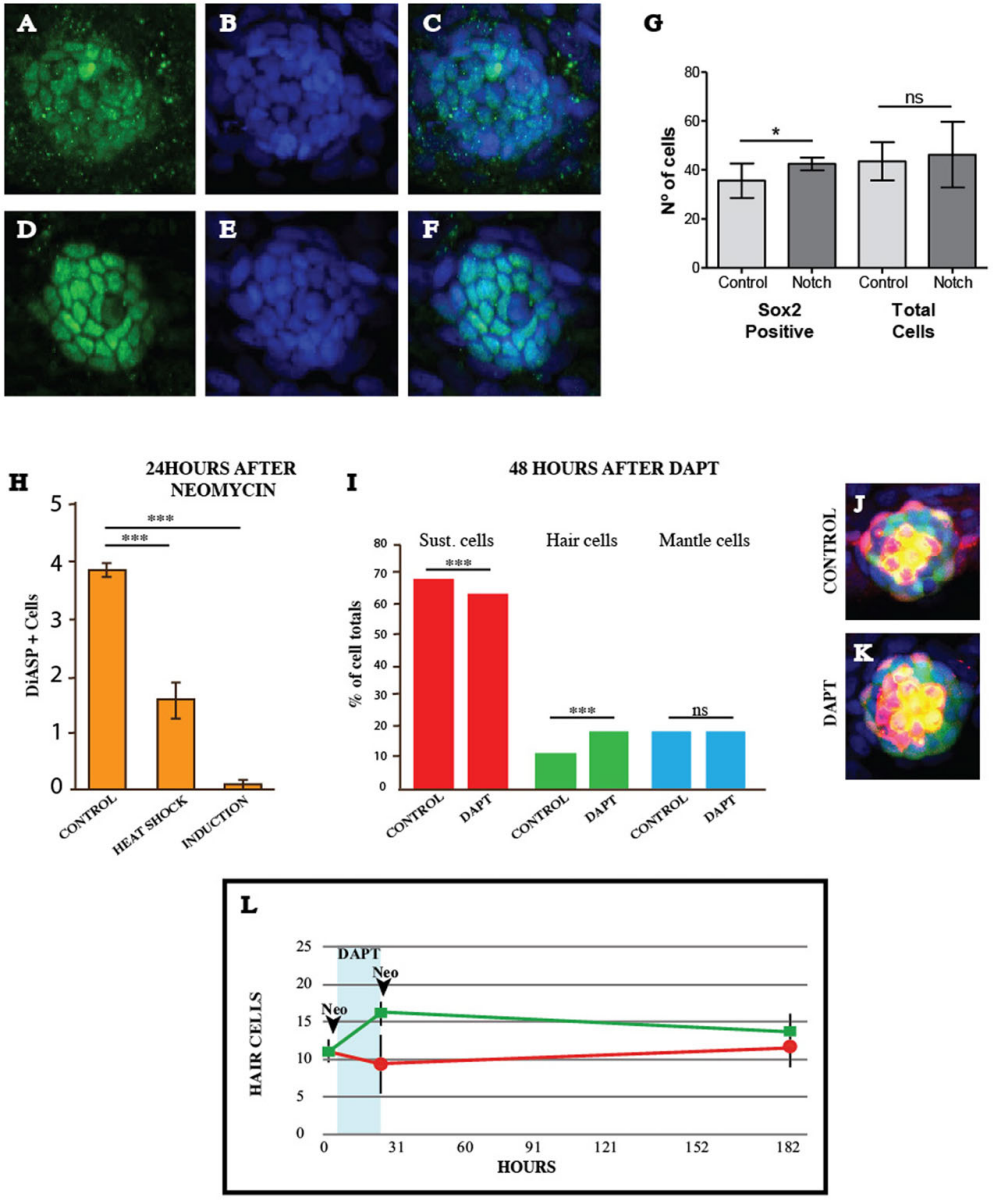
**Neuromast organization after recurrent damage.** (A-F) A neuromast immunostained for Sox-2 (red) and labeled with DAPI (blue) in (A-C) controls and (D-F) after induction of constitutive Notch signaling. (G) Quantification of Sox-2 positive cells and total cells number in regenerating neuromast 24 h after neomycin treatment with and without induction of NICD in the double transgenic line Tg[Cldnb:Gal4ERT2;5xUAS-E1b:6xMYC-notch1a], results are mean±s.e.m. (Mann-Whitney test *U*=110, Control *N*=16 (3 larvae) and NIND=23 (4 larvae), *P=0.03). (H) Quantification of regenerated hair cells 24 hpt after constitutive Notch activation by heat shock and chemical induction, results are mean±s.e.m. (Control versus Heat shock Mann-Whitney *U*=7.5, *P*<0.0001. Control versus Induction Mann-Whitney *U*=0, *P*<0.0001. Control *N*=33 (8 larvae), Heat shock *N*=7 (1 larvae), Induction *N*=14 (3 larvae) (I) Quantification of sustentacular, hair and mantle cells in regenerating neuromasts after two consecutive neomycin treatments, intercalated with a DAPT incubation period, results are mean±s.e.m. [Sust. cells Mann-Whitney *U*=61.5, *P*=0.0052. Hair cells Mann-Whitney *U*=34.5, *P*<0.0001. Mantle cells Mann-Whitney *U*=135, *P*=0.8081. Control *N*=22 (4 larvae), DAPT *N*=15 (3 larvae)]. (J-K) Representative neuromasts of the triple transgenic line Tg[ET(krt4:EGFP)sqgw57A ; Alpl:mCherry ; pou4f3: Kusabira-CAAX] used for quantifications. **(**L) Graph with hair-cell counts in regenerating neuromasts of *Tg[ET(krt4:EGFP)sqet4]* fish during 7 days, showing controls (red) and DAPT-treated fish (green). DAPT incubation period is indicated by the blue vertical bar. Results are mean±s.d. Time points: 0 h *N*=4 neuromasts (4 animals), 24 h *N*=5 neuromasts (5 animals), 182 h *N*=9 neuromasts (9 animals).

Hair cells require Atoh1a for their development. Ma and colleagues showed that Atoh1a is expressed in broad areas of the epithelium during the first 24 h of regeneration (Ma et al., 2008). Using the double-transgenic line *Tg[Atoh1a:dTomato ; ET(krt4:EGFP)sqet4]* to simultaneously visualize Atoh1a expression and hair cells, we extended these previous observations by finding a broad but weak Atoh1a in supporting cells, which stabilizes and becomes stronger in UHCPs and in young hair cells (Fig. 3E). This pattern of Atoh1a expression substantiates the prediction that neuromasts may use a large pool of supporting cells to generate UHCPs to sustain regeneration upon recurrent severe damage, and suggests that the expression of Atoh1a occurs by default in Sox-2^+^ cells. To further test this hypothesis we abrogated Notch signaling in the entire regenerating neuromast by treating zebrafish larvae with the γ-secretase inhibitor DAPT (Ma et al., 2008; Wibowo et al., 2011). The uniform loss of Notch signaling stabilized Atoh1a in a broader area of the neuromast (Fig. 3F), and generated supernumerary hair cells ectopically (Fig. 3F-G, supplementary material Fig. S2A-B). The spatial profile of Sox-2 expression and of Notch activity in neuromasts suggests that they do not regulate each other (Hernández et al., 2007; Ma et al., 2008; Wibowo et al., 2011). Supporting this conclusion, Sox-2 expression was not qualitatively affected upon a constitutive activation of Notch signaling by expressing the intracellular domain of Notch (N^ICD^) by neuromast-specific chemical induction with tamoxifen using the double transgenic line *Tg[Cldnb:Gal4^ERT2^;5xUAS-E1b:6xMYC-notch1a]* (Fig. 4A-G). However, we observed a modestly significant increase of the number of Sox-2^+^ cells that was accompanied by a small but non-significant increase of the total number of cells (Fig. 4G).

During otic development, Sox-2 establishes pro-sensory competence in the neuroepithelium for the generation of neurons and hair cells by: (1) the direct activation of Atoh1 expression and, (2) the activation of negative regulators of Atoh1 function (Neves et al., 2013). In neuromasts, however, two parallel direct inputs appear to control the spatiotemporal mode of Atoh1a expression: a constitutive and un-patterned activation by Sox-2, and a patterned inhibition by Notch. In contrast to the ear, the neuromast epithelium does not produce neurons, which may account for the differences between the two organs. The profile of Atoh1a expression in normal circumstances and in conditions of high and low Notch signaling provides a mechanistic explanation for the sequence and spatiotemporal pattern of sustentacular-cell conversion into UHCP: the mitotic division of Sox-2^+^ cells is symmetric with regard to the UHCP potential of the two daughter cells. However, the independent movement of the daughter cells within the epithelium places them in different signaling environments: the cell remaining in an equatorial zone will continue to be exposed to high levels of Notch signaling and maintain a sustentacular-cell character, whereas those entering a polar zone with low Notch activity will stabilize Atoh1a expression and become UHCP. Although we do not know if the movement of each daughter cell is stochastic or deterministic, a default broad expression of Atoh1a provides a robust mechanism for UHCP production regardless of how cells move in the tissue. These results reinforce the conclusion that potentially all Sox-2^+^ cells may quickly generate UHCPs upon reduction of Notch activity, and may explain the inexhaustible production of hair cells upon recurrent severe damages.

### Neuromast organization is reversibly affected by imbalances of Notch signaling

**Fig. 5.**
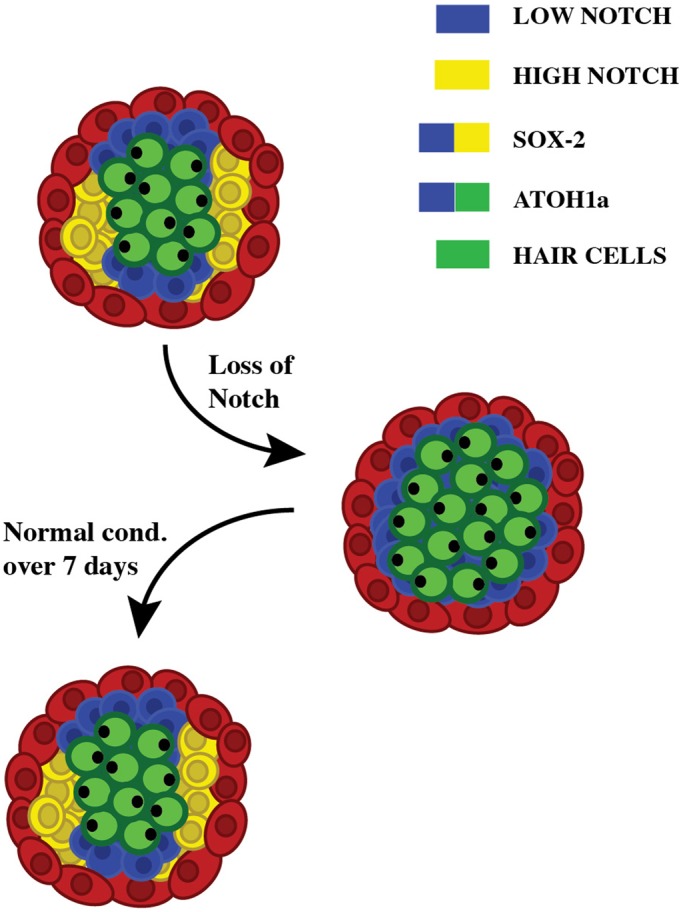
**Model of neuromast self-organization.** Schematic representation of the current model of epithelial self-organization during hair-cell regeneration, depicting the status of supporting cells upon Notch inhibition and after recovery.

It has been proposed that a feedback inhibition of supporting-cell proliferation via Notch signaling maintains a constant cellular population in neuromasts (Ma et al., 2008). Thus, to test a functional link between intercellular communication and organ proportions, we again examined neuromasts with defective Notch activity. We first expressed N^ICD^ constitutively by heat-shock in *Tg[hsp70l:Gal4; 5xUAS-E1b:6xMYC-notch1a]*, and by chemical induction with tamoxifen in the transgenic line *Tg[Cldnb:Gal4^ERT2^;5xUAS-E1b:6xMYC-notch1a],* which halted hair-cell regeneration almost immediately (Fig. 4H, supplementary material Fig. S2C), indicating that neuromasts do not contain “engaged” UHCPs that are refractory to Notch inhibition. In a converse experiment, we abrogated Notch signaling by treating zebrafish with DAPT, which increased hair-cell production (Figs 3G, 4I, supplementary material Fig. S2A-B). Total cell counts varied only marginally in DAPT-treated neuromasts and matched the excess of hair cells (on average, *circa* 16% of the cells in control neuromasts were hair cells, versus 27% in DAPT-treated samples), revealing an imbalance of cell types and epithelial proportions (Fig. 4G,I-L). Control and DAPT-treated fish were then subject to a final incubation in neomycin to ablate hair cells, and subsequently returned to normal conditions. The neuromasts that had been exposed to DAPT continued to produce supernumerary hair cells one day after the second neomycin treatment (Fig. 4L). This result suggests that after lifting Notch inhibition, a larger than normal pool of sustentacular cells produces UHCPs, possibly by a lasting re-organization of the spatial influence of Notch. Remarkably, however, these neuromasts recovered almost normal proportions one week later (Fig. 3G). Taken together, these results strongly suggest that the neuromast epithelium has self-organizing properties, which may have evolved to allow fish to maintain organ proportions during growth and repair (Fig. 5).

Collectively, the results shown above suggest that compartmentalized Notch signaling determines the geometric order of hair-cell regeneration. Thus, it will be important to discover what controls the spatiotemporal activation of the Notch receptor. One possibility is that polarized activating signals emanate from the hair cells. This could also explain the seemingly contradictory observations that although the expression of a Notch receptor is up-regulated in broad areas of the neuromast epithelium during the first 24 h after hair-cell ablation, it becomes co-expressed with Atoh1a and does not repress hair-cell production (Ma et al., 2008). Analyzing neuromasts chronically devoid of hair cells will clarify this issue.

## CONCLUSIONS

During organ repair, the number and spatial distribution of different cells must be tightly coordinated. Similarly to the *Drosophila* ovary and the mammalian skin (Blanpain and Fuchs, 2014; Xie and Spradling, 2000), neuromast regions that allow progenitor cells to progress into terminal differentiation are spatially fixed, resulting in the preferential production of hair cells in permissive polar areas. However, a single change in intercellular signaling can route a large pool of sustentacular cells into acquiring a progenitor fate, resulting in the ectopic production of supernumerary hair cells. The resulting imbalance of tissue proportions is reversible, however, supporting the notion that distinct neuromast regions are territories, as opposed to compartments, because they contain plastic cellular identities that are dynamically maintained by continuous intercellular signaling. In that, the neuromast is more reminiscent to the *Drosophila* intestine, where Notch signaling generates plastic stem-cell niches that promote localized regeneration (Mathur et al., 2010). In sum, here we present evidence that epithelial self-organization underlies the non-deterministic acquisition of UHCP fate, which governs the inexhaustible hair-cell regeneration of the lateral line.

## MATERIALS AND METHODS

### DNA constructs

The plasmid Cldnb::ERT2-GAL4 that was used to generate the stable transgenic line was placed in a miniTol2 backbone and was a gift of D. Wilkinson (NIRM, UK). To generate *Tg[Cldnb:Gal4^ERT2^]* line, 20 pg of the Tol2-expression clone and 20 pg of the transposase synthetic RNA were simultaneously injected into one-cell stage wild-type eggs. The resulting embryos were raised to adulthood and out-crossed for visual screening of germ-line transmission of the transgene.

### Zebrafish strains and husbandry

Zebrafish were maintained under standardized conditions and experiments were conducted in accordance with protocols approved by the Ethical Committee of Animal Experimentation of the Parc de Recerca Biomèdica de Barcelona (PRBB), Spain. The *ET(krt4:EGFP)sqgw57A* line was generated by random integration of an gene-trap construct (Kondrychyn et al., 2011). The following transgenic lines were described previously: *Tg[ET(krt4:EGFP)sqet4]*, *Tg[ET(krt4:EGFP)sqet20]* (Parinov et al., 2004), *Tg[Cldnb:mem-EGFP]* (Haas and Gilmour, 2006), *Tg[pou4f3:gap43-GFP]* (Xiao et al., 2005), *Tg[hsp70l:Gal4], Tg[5xUAS-E1b:6xMYC-notch1a]* (Scheer et al., 2001), *Tg[myo6b:actb1-EGFP]* (Kindt et al., 2012), and *Tg[Alpl:mCherry]* (Steiner et al., 2014), *Tg[pou4f3:Ribeye-Kusabira]* (Pujol-Martí et al., 2014).

### Heat-shock induction of gene expression

Transgenic fish carrying the hsp70 promoter were incubated at the desired developmental stage at 39°C for 30 min in groups of 30–40 embryos in 2 ml Eppendorf tubes containing a total volume of 1 ml embryo medium (Danieau 30%). Upon incubation embryos were returned to petri dishes containing embryo medium fish medium at 28.5°C where they recovered until the required time point.

### Pharmacology

Neomycin treatments were performed on 5–6 days post fertilization (dpf) transgenic zebrafish larvae or 6- to 2-year old adult fish. Neomycin sulfate at 10 mg/ml stock solution in dH_2_O (Sigma, St. Louis, MO USA), was diluted in system water to a final concentration of 250 µM. Larvae were incubated with neomycin solution for 1 h at RT, rinsed and allowed to recover in system water at 28.5°C. Sibling fish in system water at RT for 1 h and then transferred at 28.5°C served as control. Notch signaling was inhibited using the γ-secretase inhibitor DAPT (N-[N-(3,5-difluorophenacetyl)-L-alanyl]-S-phenylglycine-t-butyl ester) (Sigma). DAPT was reconstituted in 10% DMSO to a stock concentration of 150 mM and then diluted to a final concentration of 50 μM solution of DAPT in system water with 1% DMSO. Water with 1% DMSO served as control. For chemical induction of Gal4, 4-hydroxy-tamoxifen (H7904, Sigma) was dissolved at 12.5 mg/ml in 100% ethanol and stored at −20°C. Subsequent dilutions in embryo medium were made prior to use to a final concentration of 10 μM. Control larvae were incubated with an equivalent amount of ethanol diluted in embryo medium.

### Vital imaging of zebrafish larva

For vital imaging, zebrafish larvae at 6 dpf were anesthetized with a 610 µM solution of the anaesthetic 3-aminobenzoic acid ethyl ester (MS-222). Mechanoreceptive hair cells were identified by labeling with the vital dye Di-2-ASP (Molecular Probes, Eugene, OR USA) or by EGFP expression in transgenic fish. Samples were mounted onto a glass-bottom 3 cm Petri dish (MatTek, Ashland, MA USA) and covered with 1% low-melting-point agarose with diluted anaesthetic. Images were acquired with an inverted confocal microscope with a 40× air or 63× water-immersion objective lenses.

### Imaging adult caudal fins

For imaging caudal-fin neuromasts, adult zebrafish were anesthetized with a 610 µM solution of MS-222 and caudal fins were cut with a sharp scalpel. Fish were quickly returned to a tank with system water and allowed to fully recover before being transferred to a running-water system. Cut fins were fixed at 4°C in a solution of 4% paraformaldehyde (PFA) in phosphate-buffered saline (PBS) containing 0.2% Tween-20 (PBST). After fixation, the fins were washed in the same solution without fixative and transferred to a solution of Vectashield with DAPI (Vector Laboratories, Peterborough, UK) for at least one hour before imaging.

### Immunohistochemistry

Immunohistochemistry was done as described previously (Pujol-Martí et al., 2012). In brief, samples were fixed overnight at 4°C in a solution of 4% paraformaldehyde (PFA) in phosphate-buffered saline (PBS) containing 0.2% Tween-20. After fixation, the samples were washed with PBS containing 1% Tween-20 and permeabilized by incubation in acetone at −20°C for 8 min. Samples were washed with PBS 1% tween-20 and blocked at room temperature with 10% bovine serum albumin. Primary antibody was incubated for 48 h at 4°C in PBS with 0.2% Tween-20 and secondary antibody was incubated overnight at 4°C in PBS with 0.2% Tween-20. Antibodies were used at the following dilutions: rabbit anti-Sox-2 (AbCam, Cambridge, UK) at 1:200. Texas Red-labeled donkey anti-rabbit immunoglobin secondary antibody (Molecular Probes, Life Technologies, Paisley, UK) and Alexa Fluor^®^ 488 goat anti-rabbit immunoglobin secondary antibodies (A5040, Sigma) at 1:500. Images were obtained using a confocal microscope (LSM 510; Carl Zeiss).

### Quantification of hair cells

To quantify hair cells, Tg[ET(krt4:EGFP)sqet4] zebrafish larvae were used. Confocal stacks of neuromasts were acquired using an LSM 510 Zeiss microscope. Hair cells were manually identified by expression of cytoplasmic GFP. All data was processed and analyzed using a non-parametric test Mann-Whitney *U*-test (*P*<0.05, two-tailed) with GraphPad Prism version 6.04 for Windows (GraphPad Software, La Jolla, CA USA, www.graphpad.com).

## Supplementary Material

Supplementary Material

## References

[BIO012112C1] Behra M., Bradsher J., Sougrat R., Gallardo V., Allende M. L. and Burgess S. M. (2009). Phoenix is required for mechanosensory hair cell regeneration in the zebrafish lateral line. *PLoS Genet.* 5, e1000455 10.1371/journal.pgen.1000455 19381250 PMC2662414

[BIO012112C2] Blanpain C. and Fuchs E. (2014). Plasticity of epithelial stem cells in tissue regeneration. *Science* 344, 1242281 10.1126/science.1242281 24926024 PMC4523269

[BIO012112C3] Burns J. C. and Corwin J. T. (2013). A historical to present-day account of efforts to answer the question: what puts the brakes on mammalian hair cell regeneration? *Hear. Res.* 297, 52-67. 10.1016/j.heares.2013.01.005 23333259 PMC3594491

[BIO012112C4] Chen G.-D. and Fechter L. D. (2003). The relationship between noise-induced hearing loss and hair cell loss in rats. *Hear. Res.* 177, 81-90. 10.1016/S0378-5955(02)00802-X 12618320

[BIO012112C5] Ciba-Foundation, (1991). *Regeneration of Vertebrate Sensory Receptor Cells*. Chichester, UK: John Wiley & Sons.

[BIO012112C6] Groves A. K., Zhang K. D. and Fekete D. M. (2013). The genetics of hair cell development and regeneration. *Annu. Rev. Neurosci.* 36, 361-381. 10.1146/annurev-neuro-062012-170309 23724999 PMC3773239

[BIO012112C7] Haas P. and Gilmour D. (2006). Chemokine signaling mediates self-organizing tissue migration in the zebrafish lateral line. *Dev. Cell* 10, 673-680. 10.1016/j.devcel.2006.02.019 16678780

[BIO012112C8] Harris J. A., Cheng A. G., Cunningham L. L., MacDonald G., Raible D. W. and Rubel E. W. (2003). Neomycin-induced hair cell death and rapid regeneration in the lateral line of zebrafish (*Danio rerio*). *JARO* 4, 219-234. 10.1007/s10162-002-3022-x 12943374 PMC3202713

[BIO012112C9] Hernández P. P., Olivari F. A., Sarrazín A. F., Sandoval P. C. and Allende M. L. (2007). Regeneration in zebrafish lateral line neuromasts: expression of the neural progenitor cell marker Sox2 and proliferation-dependent and-independent mechanisms of hair cell renewal. *Dev. Neurobiol.* 67, 637-654. 10.1002/dneu.20386 17443814

[BIO012112C10] Huth M. E., Ricci A. J. and Cheng A. G. (2011). Mechanisms of aminoglycoside ototoxicity and targets of hair cell protection. *Int. J. Otolaryngol.* 2011, 937861 10.1155/2011/937861 22121370 PMC3202092

[BIO012112C11] Jiang L., Romero-Carvajal A., Haug J. S., Seidel C. W. and Piotrowski T. (2014). Gene-expression analysis of hair cell regeneration in the zebrafish lateral line. *Proc. Natl. Acad. Sci. USA* 111, E1383-E1392. 10.1073/pnas.1402898111 24706903 PMC3986165

[BIO012112C12] Kiernan A. E., Pelling A. L., Leung K. K. H., Tang A. S. P., Bell D. M., Tease C., Lovell-Badge R., Steel K. P. and Cheah K. S. E. (2005). Sox2 is required for sensory organ development in the mammalian inner ear. *Nature* 434, 1031-1035. 10.1038/nature03487 15846349

[BIO012112C13] Kindt K. S., Finch G. and Nicolson T. (2012). Kinocilia mediate mechanosensitivity in developing zebrafish hair cells. *Dev. Cell* 23, 329-341. 10.1016/j.devcel.2012.05.022 22898777 PMC3426295

[BIO012112C14] Kondrychyn I., Teh C., Garcia-Lecea M., Guan Y., Kang A. and Korzh V. (2011). Zebrafish Enhancer TRAP transgenic line database ZETRAP 2.0. *Zebrafish* 8, 181 10.1089/zeb.2011.0718 22181660

[BIO012112C15] Langer T., am Zehnhoff-Dinnesen A., Radtke S., Meitert J. and Zolk O. (2013). Understanding platinum-induced ototoxicity. *Trends Pharmacol. Sci.* 34, 458-469. 10.1016/j.tips.2013.05.006 23769626

[BIO012112C16] López-Schier H. and Hudspeth A. J. (2006). A two-step mechanism underlies the planar polarization of regenerating sensory hair cells. *Proc. Natl. Acad. Sci. USA* 103, 18615 10.1073/pnas.0608536103 17124170 PMC1656970

[BIO012112C17] Lush M. E. and Piotrowski T. (2014). Sensory hair cell regeneration in the zebrafish lateral line. *Dev. Dyn.* 243, 1187-1202. 10.1002/dvdy.24167 25045019 PMC4177345

[BIO012112C18] Ma E. Y., Rubel E. W. and Raible D. W. (2008). Notch signaling regulates the extent of hair cell regeneration in the zebrafish lateral line. *J. Neurosci.* 28, 2261-2273. 10.1523/JNEUROSCI.4372-07.2008 18305259 PMC6671837

[BIO012112C19] Mathur D., Bost A., Driver I. and Ohlstein B. (2010). A transient niche regulates the specification of Drosophila intestinal stem cells. *Science* 327, 210-213. 10.1126/science.1181958 20056890 PMC2857772

[BIO012112C20] Millimaki B. B., Sweet E. M. and Riley B. B. (2010). Sox2 is required for maintenance and regeneration, but not initial development, of hair cells in the zebrafish inner ear. *Dev. Biol.* 338, 262-269. 10.1016/j.ydbio.2009.12.011 20025865 PMC2815045

[BIO012112C21] Neves J., Vachkov I. and Giraldez F. (2013). Sox2 regulation of hair cell development: incoherence makes sense. *Hear. Res.* 297, 20-29. 10.1016/j.heares.2012.11.003 23154195

[BIO012112C22] Parinov S., Kondrichin I., Korzh V. and Emelyanov A. (2004). Tol2 transposon-mediated enhancer trap to identify developmentally regulated zebrafish genes in vivo. *Dev. Dyn.* 231, 449-459. 10.1002/dvdy.20157 15366023

[BIO012112C23] Pinto-Teixeira F., Muzzopappa M., Swoger J., Mineo A., Sharpe J. and López-Schier H. (2013). Intravital imaging of hair-cell development and regeneration in the zebrafish. *Front. Neuroanat.* 7, 33 10.3389/fnana.2013.00033 24130521 PMC3795300

[BIO012112C24] Pujol-Martí J., Zecca A., Baudoin J.-P., Faucherre A., Asakawa K., Kawakami K. and López-Schier H. (2012). Neuronal birth order identifies a dimorphic sensorineural map. *J. Neurosci.* 32, 2976-2987. 10.1523/JNEUROSCI.5157-11.2012 22378871 PMC6622018

[BIO012112C25] Pujol-Martí J., Faucherre A., Aziz-Bose R., Asgharsharghi A., Colombelli J., Trapani J. G. and López-Schier H. (2014). Converging axons collectively initiate and maintain synaptic selectivity in a constantly remodeling sensory organ. *Curr. Biol.* 24, 2968-2974. 10.1016/j.cub.2014.11.012 25484295

[BIO012112C26] Rubel E. W., Furrer S. A. and Stone J. S. (2013). A brief history of hair cell regeneration research and speculations on the future. *Hear. Res.* 297, 42-51. 10.1016/j.heares.2012.12.014 23321648 PMC3657556

[BIO012112C27] Scheer N., Groth A., Hans, and Campos-Ortega J. A. (2001). An instructive function for Notch in promoting gliogenesis in the zebrafish retina. *Development* 128, 1099-1107. 11245575 10.1242/dev.128.7.1099

[BIO012112C28] Schuck J. B. and Smith M. E. (2009). Cell proliferation follows acoustically-induced hair cell bundle loss in the zebrafish saccule. *Hear. Res.* 253, 67-76. 10.1016/j.heares.2009.03.008 19327392 PMC2810637

[BIO012112C29] Steiner A. B., Kim T., Cabot V. and Hudspeth A. J. (2014). Dynamic gene expression by putative hair-cell progenitors during regeneration in the zebrafish lateral line. *Proc. Natl. Acad. Sci. USA* 111, E1393-E1401. 10.1073/pnas.1318692111 24706895 PMC3986164

[BIO012112C30] Wibowo I., Pinto-Teixeira F., Satou C., Higashijima S.-i. and López-Schier H. (2011). Compartmentalized Notch signaling sustains epithelial mirror symmetry. *Development* 138, 1143-1152. 10.1242/dev.060566 21343366

[BIO012112C31] Williams J. A. and Holder N. (2000). Cell turnover in neuromasts of zebrafish larvae. *Hear. Res.* 143, 171-181. 10.1016/S0378-5955(00)00039-3 10771194

[BIO012112C32] Xiao T., Roeser T., Staub W. and Baier H. (2005). A GFP-based genetic screen reveals mutations that disrupt the architecture of the zebrafish retinotectal projection. *Development* 132, 2955-2967. 10.1242/dev.01861 15930106

[BIO012112C33] Xie T. and Spradling A. C. (2000). A niche maintaining germ line stem cells in the *Drosophila* ovary. *Science* 290, 328-330. 10.1126/science.290.5490.328 11030649

