# Supplementary material for: Inexhaustible hair-cell regeneration in young and aged zebrafish
Source: Biol Open. 2015 May 22;4(7):903–9. doi: 10.1242/bio.012112 (PMC4571094; doi:10.1242/bio.012112)
Supplement: Supplementary Material [file supp_4_7_903__index.html]

Inexhaustible hair-cell regeneration in young and aged zebrafish — Inexhaustible hair-cell regeneration in young and aged zebrafish — Supplementary Material 

# Inexhaustible hair-cell regeneration in young and aged zebrafish

## BIO012112 Supplementary Material

- Supplementary Material
