# Supplementary material for: Inexhaustible hair-cell regeneration in young and aged zebrafish
Source: Biol Open. 2015 May 22;4(7):903–9. doi: 10.1242/bio.012112 (PMC4571094; doi:10.1242/bio.012112)
Supplement: Supplementary Material [file supp_bio.012112_BIO012112supp.pdf]

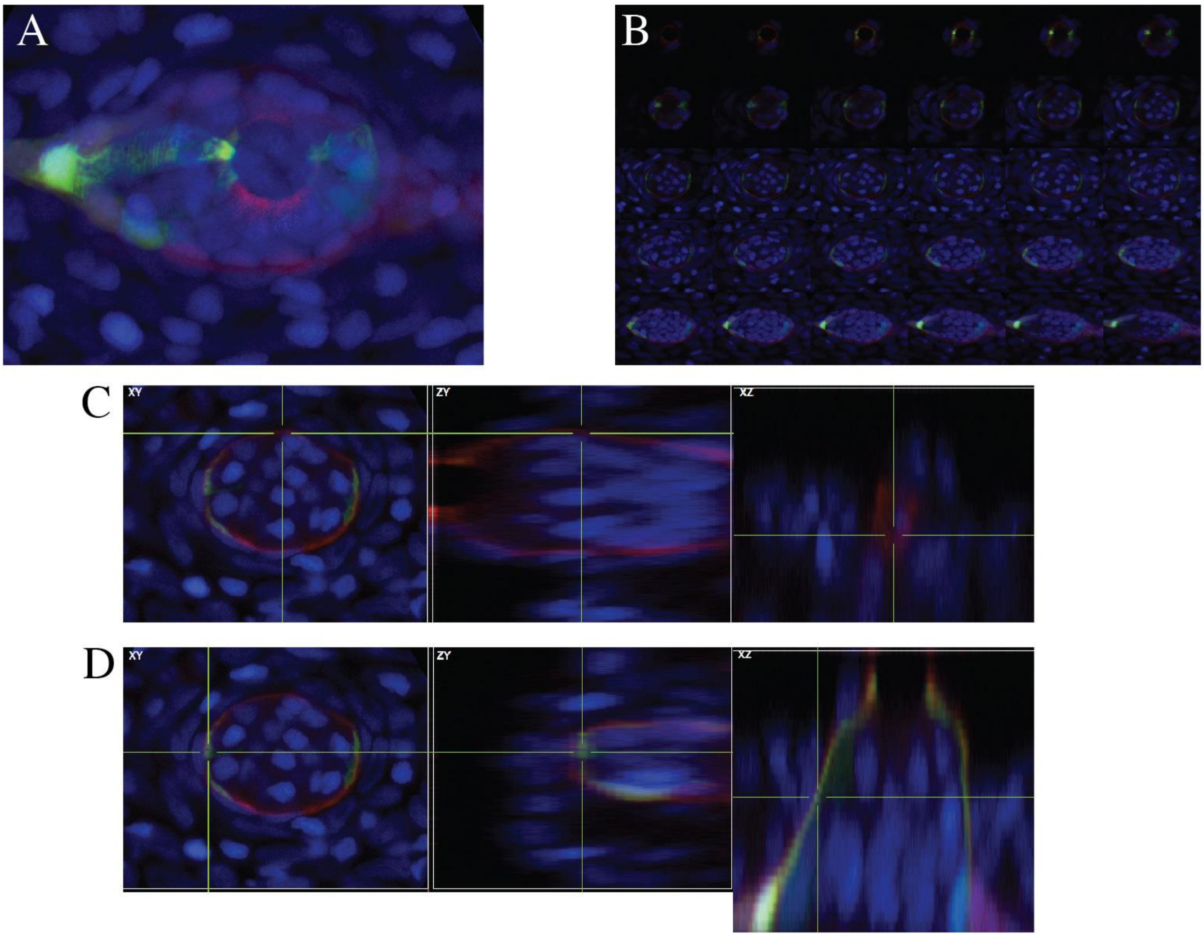

Fig. S1. (A-D) A neuromast from an adult fish of the double transgenic line *Tg[ET(krt4:EGFP)sqet20 (green); Alpl:mCherry (red)]* counterstained with DAPI (blue), highlighting mantle cells (red) and the equatorial areas (green). (A) shows a maximal projection from an apical-to-basal Z-stack. (B) shows the series of individual stacks. (C) shows a single stack at a polar area, and (D) shows a single stack at an equatorial area. They reveal that *Tg[ET(krt4:EGFP)sqet20]* marks preferentially the equatorial areas.

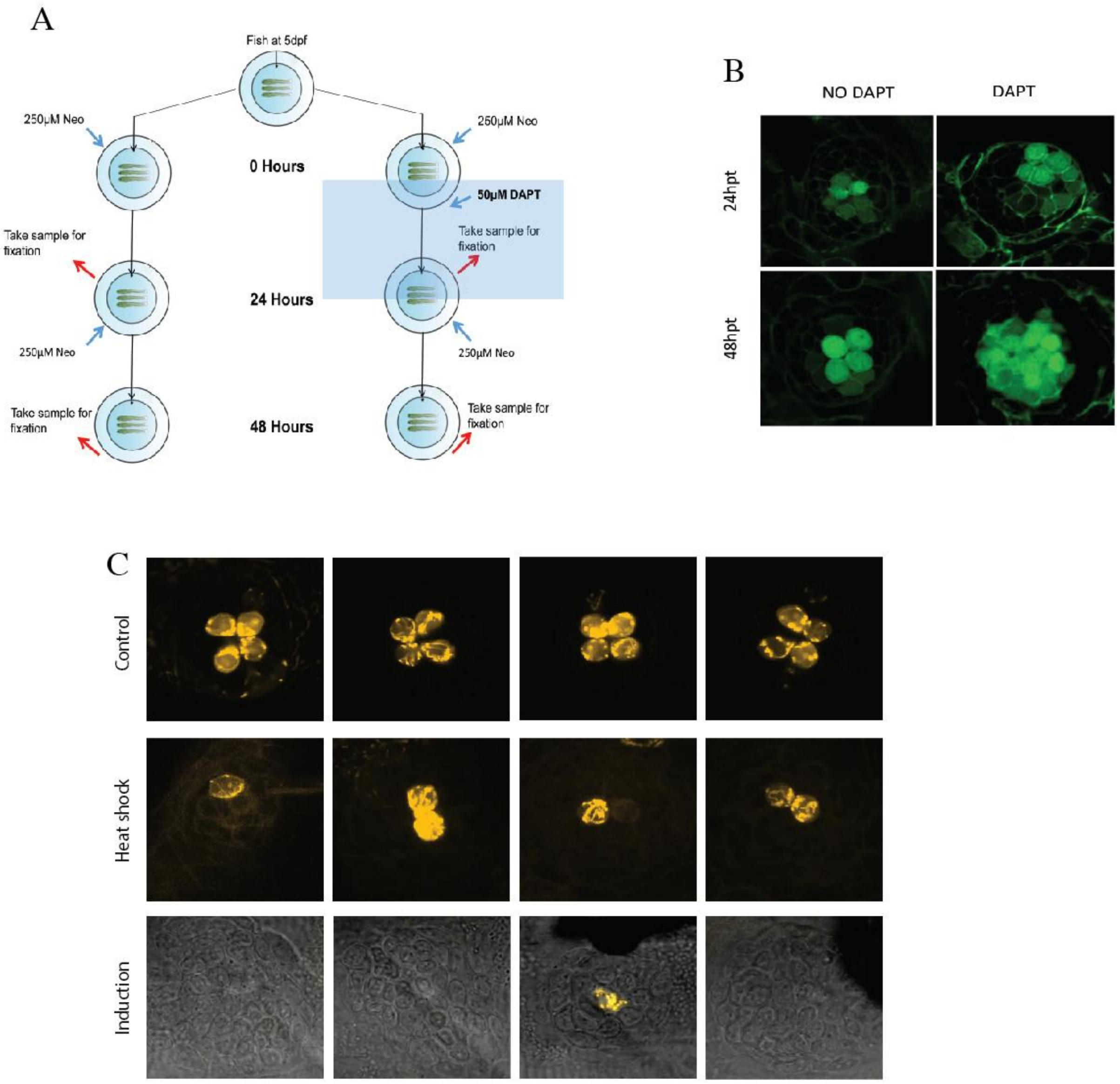

Fig. S2. (A) A scheme showing the protocol for neomycin and DAPT treatments and hair-cell quantifications during regeneration. (B) representative neuromasts of *Tg[ET(krt4:EGFP)sqet4 ; Cldnb:mem-EGFP]*, revealing hair cells 24 and 48 hpt, with and without DAPT treatments. (C) shows a series of neuromasts that reveal hair cells with DiASP labeling 24 hpt, after constitutive Notch activation by heat shock in the double transgenic line *Tg[hsp70l:Gal4 ; 5xUAS-E1b:6xMYC-notch1a]*, and by chemical induction with tamoxifen in the transgenic line *Tg[Cldnb:Gal4ERT2;5xUAS-E1b:6xMYC-notch1a]*.
